# The Only Antidote to the Bane of Whiskey*Thomas Jefferson’s *Writings*, vol. XV, p. 178.

**Published:** 1913-12

**Authors:** 


### EDITORIAL DEPARTMENT.

**DR. F. E. DANIEL, Editor** **DR. R. H. L. BIBB, Associate Editor**

DEPARTMENTS { EUGENICS: Drs. M. Duggan and T. Y. Hull.

WOMAN'S DEPARTMENT: Mrs. F. E. Daniel.

### "The Only Antidote to the Bane of Whiskey."*

Thomas Jefferson is and always has been regarded as the little god of democracy. He is universally esteemed as one of the ablest men of this or any other age. His words and opinions have great weight in socio-political affairs today and here is what he thinks will solve the vexed liquor question. I may say in passing, that the great Adam Smith, the great authority on Social Economics in his immortal work, "Wealth of Nations," holds and expresses the same opinion. Mr. Jefferson says:

"I rejoice as a moralist, at the prospect of a reduction of the duties on wine, by our national legislature. It is an error to view a tax on that liquor as merely a tax on the rich. It is a prohibition of its use to the middling class of our citizens, and a condemnation of them to the poison of whiskey which is desolating their homes. No nation is drunken where wine is cheap; and none sober where the dearness of wine substitutes ardent spirits as the common beverage. It is, in truth, the only antidote to the bane of whiskey. Fix but the duty at the rate of other merchandise and we can drink wine * * * as cheap as we do grog. * * * Every one in easy circumstances will prefer it to the poison to which they are now driven by their government."

So much for Mr. Jefferson's opinion. He would compromise with the great drink evil—which like the deadly upas overshadows and desolates our land. As a matter of expediency, yes, perhaps it would get votes for a candidate who advocates it, but the RED BACK will never consent to sacrifice the health, the lives and the happiness of the people to a matter of expediency. In Mr. Jefferson's day they knew nothing of lager beer. Its consumption is something enormous. I suppose he would have exempted it from prohibition, also, and it would be a most acceptable compromise to the large foreign element of our population, and would be a tremendous vote-getter. Nor did Mr. Jefferson know many things about wine and beer that have since been learned in the laboratory. Both contain alcohol, more or less, and are intoxicating. That word seems never to be taken at its value. It means "poisoned."

*Thomas Jefferson's *Writings*, vol. XV, p. 178.

It is known now that alcohol taken regularly even in the small quantities represented in wine and beer is a large factor in the production of most chronic diseases, notably in Bright's disease, a malady that carries off a large percentage of middle-aged men, men who should and who probably would live fifteen or twenty years more. It is the cause of numerous diseases of the liver, cirrhosis, followed by dropsy, fatty degeneration of the liver, softening of the brain, fatty degeneration of the heart, and numerous other ills. No! no compromise with the Demon Alcohol. Pluck the evil out by the root. The war is on in Texas and in the next campaign we will elect a sensible and level-headed governor who will not veto the liquor bills as fast as a sensible Legislature passes them. There is no use in advocating "preventive medicine" so long as the State licenses Tom, Dick and Harry to poison the people with alcohol.

Pluck it out. *Prohibit its importation, manufacture and sale.*

